# Microvascular assessment of fascio-cutaneous flaps by ultrasound: A large animal study

**DOI:** 10.3389/fphys.2022.1063240

**Published:** 2022-12-15

**Authors:** Guillaume Goudot, Yanis Berkane, Eloi de Clermont-Tonnerre, Claire Guinier, Irina Filz von Reiterdank, Antonia van Kampen, Korkut Uygun, Curtis L. Cetrulo, Basak E. Uygun, Anahita Dua, Alexandre G. Lellouch

**Affiliations:** ^1^ Cardiology Division, Massachusetts General Hospital, Harvard Medical School, Boston, MA, United States; ^2^ Hôpital Européen Georges-Pompidou, Assistance Publique—Hôpitaux de Paris (APHP), Université Paris-Cité, Paris, France; ^3^ Shriners Children’s Boston, Boston, MA, United States; ^4^ Centre Hospitalier Universitaire de Rennes, Université de Rennes 1, Rennes, France; ^5^ Division of Plastic and Reconstructive Surgery, Vascularized Composite Allotransplantation Laboratory Center for Transplantation Sciences, Massachusetts General Hospital Harvard Medical School, Boston, MA, United States; ^6^ Center for Engineering in Medicine and Surgery, Massachusetts General Hospital, Harvard Medical School, Boston, MA, United States; ^7^ Division of Cardiac Surgery, Massachusetts General Hospital, Harvard Medical School, Boston, MA, United States; ^8^ University Clinic of Cardiac Surgery, Leipzig Heart Center, Leipzig, Germany; ^9^ Division of Vascular and Endovascular Surgery, Massachusetts General Hospital, Harvard Medical School, Boston, MA, United States; ^10^ Department of Plastic, Reconstructive and Aesthetic Surgery, Groupe Almaviva Santé, Clinique de l’Alma, IAOPC, Paris, France

**Keywords:** vascularity index, microvascularization, flap surgery, pig, microcirculation, Microvascular flow imaging, MV-Flow

## Abstract

**Objectives:** Blood perfusion quality of a flap is the main prognostic factor for success. Microvascular evaluation remains mostly inaccessible. We aimed to evaluate the microflow imaging mode, MV-Flow, in assessing flap microvascularization in a pig model of the fascio-cutaneous flap.

**Methods:** On five pigs, bilateral saphenous fascio-cutaneous flaps were procured on the superficial femoral vessels. A conventional ultrasound evaluation in pulsed Doppler and color Doppler was conducted on the ten flaps allowing for the calculation of the saphenous artery flow rate. The MV-Flow mode was then applied: for qualitative analysis, with identification of saphenous artery collaterals; then quantitative, with repeated measurements of the Vascularity Index (VI), percentage of pixels where flow is detected relative to the total ultrasound view area. The measurements were then repeated after increasing arterial flow by clamping the distal femoral artery.

**Results:** The MV-Flow mode allowed a better follow-up of the saphenous artery’s collaterals and detected microflows not seen with the color Doppler. The VI was correlated to the saphenous artery flow rate (Spearman rho of 0.64; *p* = 0.002) and allowed to monitor the flap perfusion variations.

**Conclusion:** Ultrasound imaging of microvascularization by MV-Flow mode and its quantification by VI provides valuable information in evaluating the microvascularization of flaps.

## 1 Introduction

Fascio-cutaneous flaps have become the gold-standard technique for complex defect reconstruction (Paro et al., 2016). By sparing the muscle while providing a vascularized skin paddle, the surgeon can cover defects near or distant from the donor site by performing pedicled or free fascio-cutaneous flaps (Cariou, 1995; Boretto and De Cicco, 2022). Assessment of segmental perfusion of a fascio-cutaneous flap is an important viability factor, although challenging in daily practice. When perfusion failure is suspected during surgery and postoperatively, a prompt assessment is mandatory to consider arterial or venous thrombosis and to allow a possible therapeutic procedure (Liu et al., 2020). Ultrasound imaging is particularly suitable because of its ease of use and ability to evaluate superficial tissue with good image quality. However, conventional Doppler modes only allow a limited assessment of flap perfusion, and most microvascularization remains inaccessible. Nevertheless, access to an evaluation of the microvascular network is a major challenge because the good capacity of the underlying microvascular network is responsible for the good perfusion of the flap. New ultrasonic methods dedicated to slow flow visualization have been recently developed to access the microvascular network, such as MV-Flow (Gettle and Revzin, 2020; Giuffrida et al., 2021). Based on a high frame rate associated with dedicated filters, MV-Flow allows a high sensitivity of flow measurements in small arterioles and venules, giving access to a vascular mapping of tissue (Aghabaglou et al., 2022).

Our objective was to evaluate the MV-Flow mode’s ability to assess flap microvascularization in a clinically relevant large animal model by evaluating its potential to detect arteriolar and venular microflows and quantify perfusion changes.

## 2 Methods

### 2.1 Experimental flap model

**FIGURE 1:**
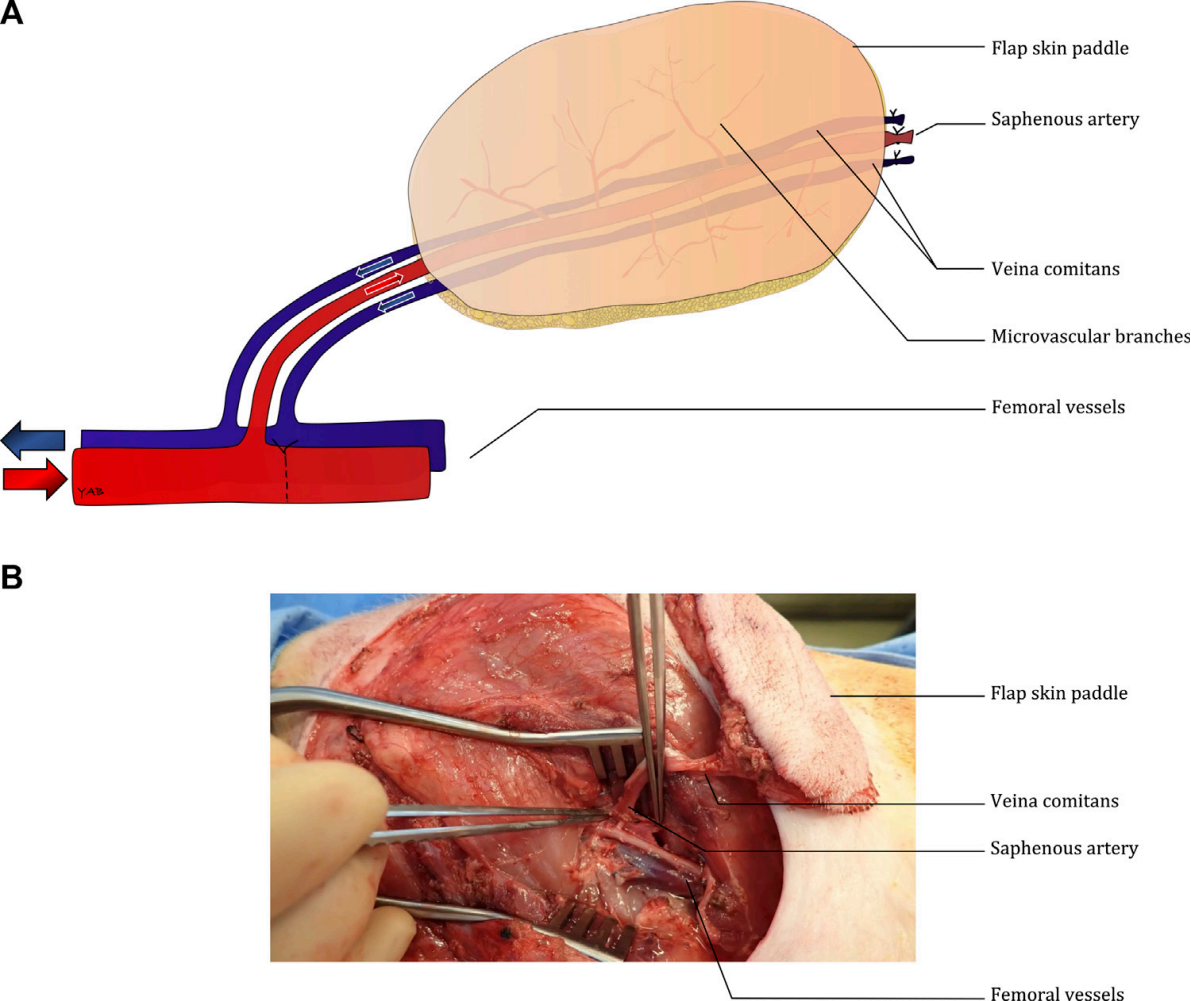
Diagram describing the surgical model **(A)** and intra-operative picture of the bilateral fasciocutaneous flaps **(B)**.

All animal care and use in the present study were approved by the Massachusetts General Hospital (Boston, MA, United States) Institutional Animal Care and Use Committee (Protocol 2020N000015). Ten saphenous fascio-cutaneous flaps were procured from five 30–35 kg Yorkshire pigs under general anesthesia. As previously described by Pozzo et al. (Pozzo et al., 2022), the distal saphenous pedicle was ligated to allow the flap to be elevated distally to proximally (Figure 1). The vessel dissection was performed up to the femoral vessels, which were subsequently dissected (Supplementary Video S1). After flap elevation, a single dose of heparin (100 UI/kg) was given intravenously before the ultrasound evaluation. The ultrasonic assessment was performed at the end of the bilateral flap harvesting. After initial ultrasound analysis, the femoral artery was clamped distally to the saphenous pedicle (Figure 1) to increase flap perfusion.

### 2.2 Ultrasound Imaging

Ultrasound examination was performed with a Samsung RS85^®^ (Samsung Medison Co., Seoul, Korea). We performed flap views with minimal compression to obtain the best microvascularization analysis using MV-Flow mode (Supplementary Video S2). The Vascularity Index (VI) corresponds to the percentage of pixels where flow is detected relative to the total ultrasound evaluation area (Chen et al., 2021; Baek et al., 2022). Three measurements of VI were performed in a standardized format during systole in a 2.0 cm^2^ region of interest, starting at the level of the superficial dermis and excluding the saphenous artery. Three measurements of VI were performed in a standardized format: measurement in systole (for small arteries, this allowed to obtain a better flow) from video back-up.

The baseline arterial flow rate was measured on the proximal saphenous artery and obtained by the average of three measures with an automated record of the mean velocity by pulsed Doppler, and measurement of the internal diameter of the artery by color Doppler, by the average of two diastolic and one systolic measurement. The flow rate in the femoral artery was also measured similarly, by three pulsed Doppler mean velocity measurements and three transverse artery diameter measurements. Intra-observer repeatability of VI measurements was calculated based on 10 VI measurements by a single observer. Sequential measurements of two observers on different ultrasonic acquisitions assessed inter-observer variations.

### 2.3 Statistical analysis

Continuous variables are presented by the median [25th–75th percentiles]. A Wilcoxon signed-rank test was used for paired data comparisons. The correlation was performed using a Spearman rank test. The interclass correlation coefficient (ICC) assessed the reliability of VI. Statistical significance was considered at the 0.05 level. Analyses were performed using R^®^ software (R-Studio, Boston, MA, United States).

## 3 Results

### 3.1 Conventional ultrasonic assessment of arterial flow in the flap

Good vascular patency was noted for all ten flaps. The mean velocity over the cardiac cycle of the saphenous artery was 1.31 cm/beat [0,84–2.43] with a flow rate of 1.13 mL/min [0.87–1.37] (Table 1). This corresponded to an average of 2.3% of the femoral artery flow rate measured at 79.7 ml/min [53.5–118.4].

**TABLE 1:** Hemodynamic analysis of the flap before (physiological flow) and after clamping the distal femoral artery. Results are presented as median [25th-75th] percentiles. *p*-values are obtained from Wilcoxon paired signed-rank test. Data in bold correspond to statistically significant comparisons (*P* < 0.05).

	Flap with physiological flow	After distal femoral artery clamping	*p*-value
Arterial diameter (mm)	1.00 [1.00–1.10]	1.20 [1.10–1.14]	0.075
Maximum velocity time integral (cm/beat)	2.58 [2.08–3.58]	4.38 [3.61–5.13]	**0.008**
Mean velocity time integral (cm/beat)	1.31 [0.84–2.43]	2.21 [1.30–2.70]	0.300
Arterial flow rate (ml/min)	1.13 [0.87–1.37]	1.98 [1.58–2.70]	**0.004**
Vascularity Index (%)	3.15 [2.48–4.38]	6.93 [4.82–8.67]	**0.002**

### 3.2 Qualitative evaluation of the MV-Flow mode: identification of microvessels

**FIGURE 2:**
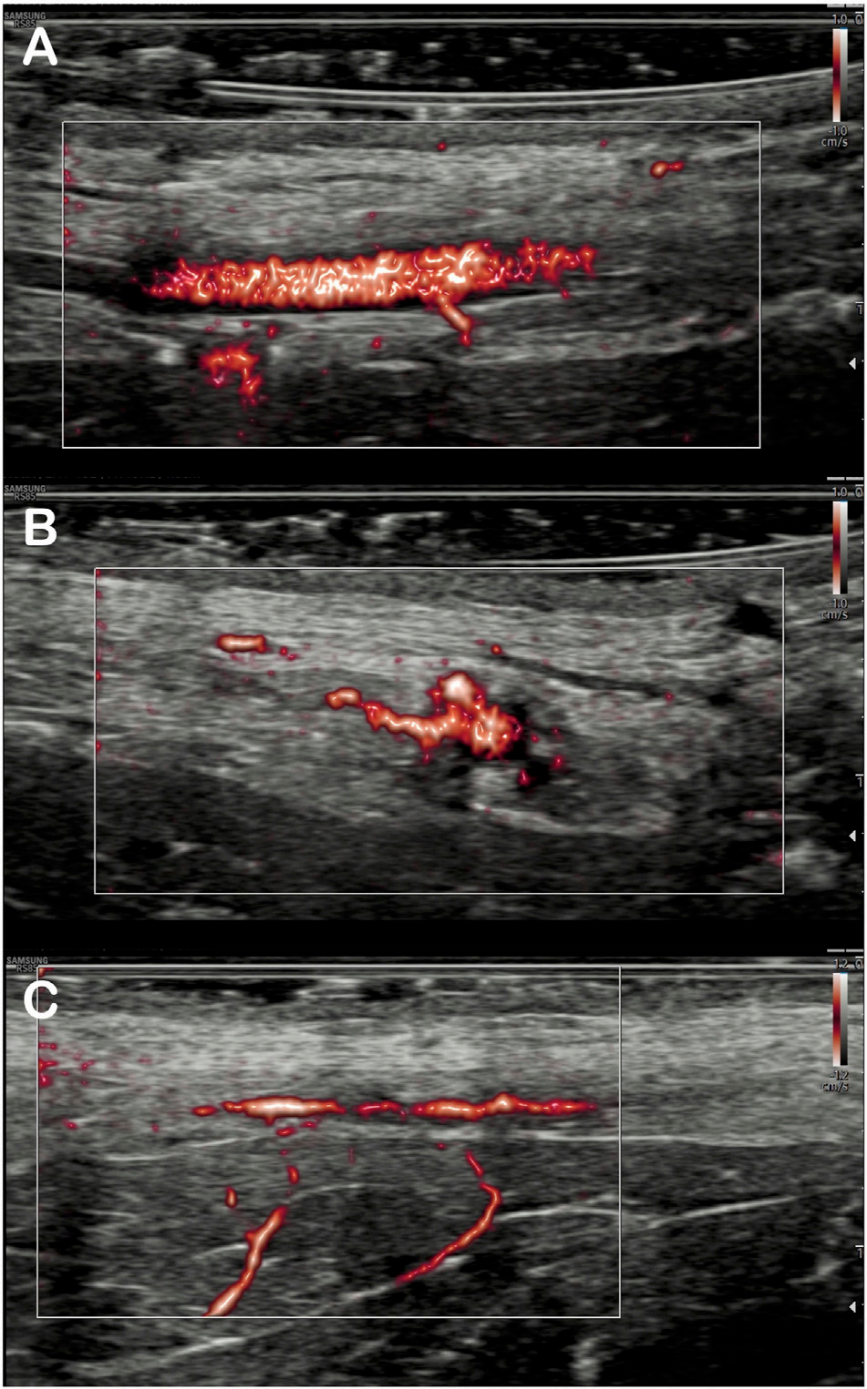
Imaging of the arterial branches of the saphenous artery by MV-flow. Detection of saphenous artery collaterals in the flap: The origin of inferior collateral is viewed in the longitudinal section **(A)**, and, for the same artery, more proximal collateral is detected in the transverse section **(B)**. Visualization of two deep inferior collaterals on another flap **(C)**.

**FIGURE 3:**
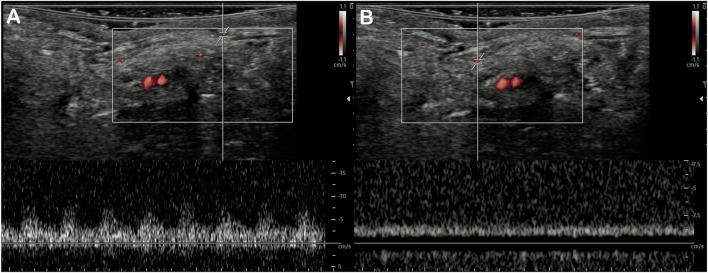
Detection of superficial microvessels by MV-Flow. The use of pulsed Doppler with a positioned recording area at the MV-Flow detection site allows differentiating small arteries with the pulsed flow **(A)** from small veins with the continuous flow **(B)** in the same field of view.

MV-Flow mode allowed better visualization of the saphenous artery trajectory than the color Doppler mode in a first qualitative analysis, with the ability to visualize the start and initial course of arterial collaterals (Figure 2). MV-Flow mode also allowed the identification of microflows on the whole thickness of the flap. The successive use of pulsed Doppler-guided by the MV-Flow allowed the authentication of the presence of small arteries or veins according to the flow pulsatility (Figure 3).

### 3.3 Quantitative evaluation by vascularity index: Detection of local perfusion changes

**FIGURE 4:**
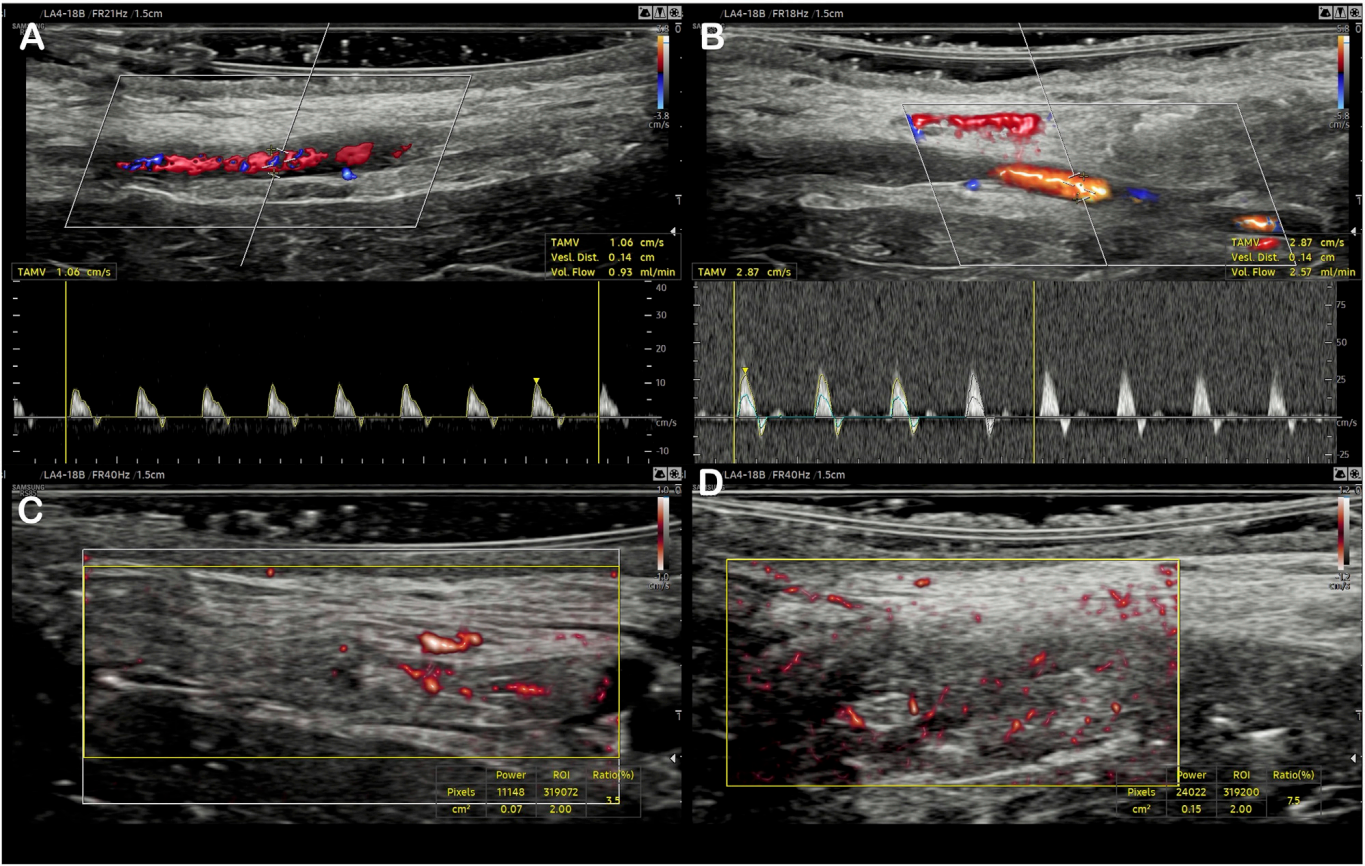
Evaluation of the initial arterial flow using color Doppler and pulsed Doppler **(A)**, then after femoral artery clamping **(C)**. A similar presentation of the Vascularity Index for the same flap using MV-Flow, at baseline **(B)**, and after flow increase **(D)**.

**FIGURE 5:**
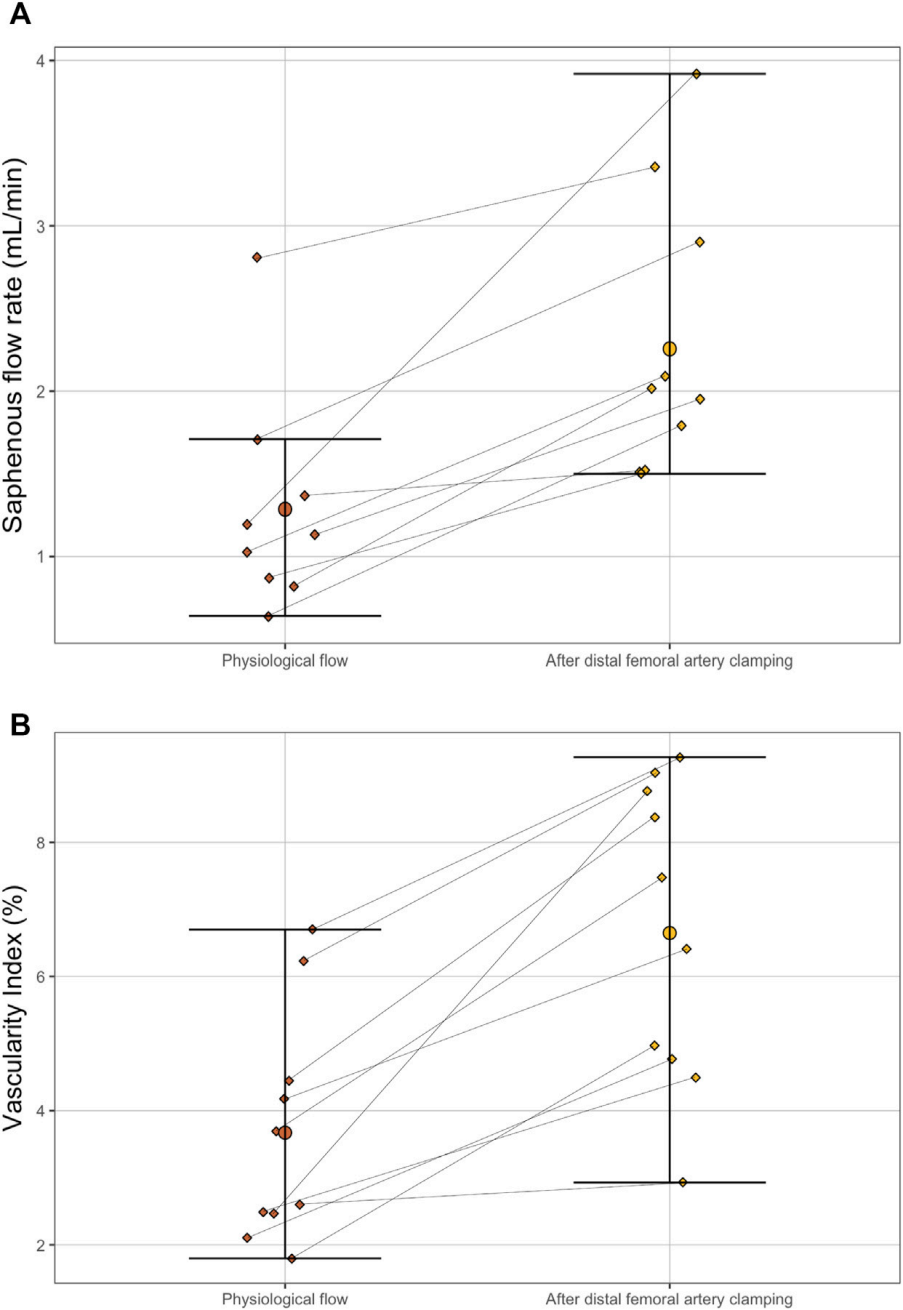
Paired dot plot of saphenous artery flow rate **(A)** and Vascularity Index **(B)** between physiological flow (before the distal femoral artery camping maneuver) and after **(B)**. The differences were significant for both parameters according to paired Wilcoxon test (*p* = 0.004 for the flow rate and *p* = 0.002 for the Vascularity Index).

The distal femoral artery clamping maneuver was associated with improved visual pulsatility of the saphenous artery and a notable increase in diameter. The flow rate increase was noted by ultrasound: 1.98 ml/min [1.58–2.70] vs 1.13 [0.87–1.37], *p* = 0.004 (Table 1). Outside the field of view of the artery, the VI was significantly increased: 6.93 [4.82–8.67] vs 3.15 [2.48–4.38], *p* = 0.002 (Table 1; Figures 4, 5). There was a good correlation between saphenous arterial flow and VI (Spearman rho of 0.64; *p* = 0.002).

### 3.4 Intra- and inter-observer reliability of Vascularity Index

Intra-observer and inter-observer ICC were respectively 0.86 and 0.95 for the VI, thus showing a good reproducibility of the measurements.

## 4 Discussion

In this work, MV-flow mode allowed the visualization of small superficial vessels and the micro vascularization quantification related to the flap perfusion, not accessible with conventional Doppler. Currently expanding, the use of methods dedicated to micro vascularization is still limited, with little validation and low accessibility of new-generation devices until now. Nevertheless, they allow for easy characterization of microflows, opening up many clinical applications (Jin et al., 2022; Tang et al., 2022).

The Vascularity Index seems particularly useful as it provides a simple and segmental quantification of vascularization. Applied to the fascio-cutaneous flap, it brings an easier detection of small arteries and veins, with a potential impact during intraoperative use (Tashiro et al., 2016). Furthermore, it allows a local estimation of the perfusion, which can be segmental, and does not require the registration of the feeding artery, which is sometimes not easily accessible in case of a deep artery or poor image quality. Lastly, this mode does not require a dedicated research device as microvascular flow imaging modes are now included in many ultrasound scanners of most of the companies (Gettle and Revzin, 2020; Aziz et al., 2022). A technology transfer to clinical practice is thus potentially achievable under the condition that such equipment will be widely accessible. Further clinical applications of microvascular flow imaging, such as the early detection of intraoperative perfusion defects, even possible by the surgeon himself, will need to be validated.

## 5 Limitations

The VI has only been used to quantify highly vascularized organs such as the thyroid (mean values of normal thyroid around 20%) or placenta (mean values of normal placenta around 45%) (Chen et al., 2021; Baek et al., 2022). We present the first evaluation on the skin, much less vascularized, thus facing the challenge of accurate signal measurement without motion artifacts. Since *in vivo* microvascular assessment technologies are limited, this study lacks comparison to a gold standard Imaging method. While no reference values are available to compare our data, we expect that our values will be used as a comparison for future studies. Doppler quantification of the arterial flow rate was performed to overcome this limitation by giving insight into global flap perfusion. Another limitation is that the VI has not been directly compared to visualization in color Doppler. A color Doppler vascularity index has been proposed for organs that are more perfused than the fascio-cutaneous flap, such as the kidney or the thyroid (Chen et al., 2002; Sultan et al., 2015; Zhang et al., 2022). In these cases, the higher blood flow velocities allowed for color mapping and area comparison with conventional Doppler. However, this was not the case for cutaneous and subcutaneous tissues where low blood flow velocities resulted in poor color Doppler visualization. Lastly, VI should be ideally measured in 3D. A perspective is the use of a 3D mode of microvascular imaging currently arising (Zhang et al., 2019; Cai et al., 2021).

## 6 Conclusion

Ultrasound imaging with MV-Flow and microflow quantification by Vascularity Index provides valuable information in evaluating flap microvascularization.

## Data Availability

The raw data supporting the conclusions of this article will be made available by the authors, without undue reservation.
